# Static telepathology in cancer institute of Tehran university: report of the first academic experience in Iran

**DOI:** 10.1186/1746-1596-1-33

**Published:** 2006-10-04

**Authors:** Afshin Abdirad, Babak Sarrafpour, Siavash Ghaderi-sohi

**Affiliations:** 1 Assistant Professor of Pathology, Cancer Institute, Tehran University of Medical Sciences, Cancer Institute, Keshavarz Blvd., Tehran, Iran; 2 Resident of Oral Pathology, School of Dentistry, Tehran University of Medical Sciences, Enghelab St, Tehran, Iran; 3 Resident of Pathology, Cancer Institute, Tehran University of Medical Sciences, Cancer Institute, Keshavarz Blvd., Tehran, Iran

## Abstract

Telepathology is the practice of pathology, which allows quick and timely access to an expert opinion at a distance. We analyzed our new experience in cancer Institute of Tehran University of Medical Sciences with the iPath telepathology server of Basel University. One hundred sixty one cases in a period of 32 months were consulted. These cases received for second evaluation but the definite diagnosis could not be made in this centre. The number of images per case ranged from 3 to 32 (mean: 8). Except one case all cases were evaluated by consultants. Definite final diagnosis was achieved in 88/160 (54.7%). Recommendations for further evaluation were offered in 42/160 cases (26%). Major discrepancies were encountered in 30/160 cases (19%). Thirty-nine of the cases (24.3%) were reported within 1 day. The rate of achieving final diagnosis was higher in histological group rather than cytological ones. Increase in number of H&E images had no significant effect on achieving a definite final diagnosis. The rate of achieving final diagnosis in this study is much lower than other similar studies, which could be due to inappropriate sampling images, a potential cause of misdiagnosis in static telepathology. The other possible reason is that all of the cases in this study were problematic cases that a definite diagnosis could not be made for them even in primary consultation. The mean time for achieving a final diagnosis was also more than other studies, which could be for the reasons mentioned above.

## Background

Telepathology is a process of histopathologic diagnosis through the digital images of both gross and microscopic findings of the specimens instead of conventional glass slides [1,2] which is usually use electronic transmission of the images to a remote centre. One of the first uses of telepathology in practice is occurred in 1973 from the ship docked in Brazil through a satellite for transmission of bone marrow smear images to Washington [3]. In 1990 the first network of telepathology was established in Norway between five hospitals without on-site pathologist for remote frozen section diagnosis [4] and till the end of 20^th ^century with significant development in computer based sciences and digital imaging the telepathology was expanded rapidly [5,6]. Nowadays telepathology is used for diagnosis (frozen section and permanent section), consultation and continual medical education.

It has two basic forms, static and dynamic. The static telepatholoy is the simplest method in telepathology with capture of digital images and then electronic transmission of them [7] and is the less effective one because of the possible sampling field errors by submitting pathologist [8]. Dynamic telepathology is real-time transmission of image from a light microscope to a distance with robotic control of the stage. Disadvantages of this method are high cost and unavailability of appropriate connection lines in many areas. A new third approach to telepathology is hybrid systems whish combine static and dynamic elements [9]. In these systems a series of static images are captured and stored, then during the time of teleconsultation or tele-education they are transmitted consequently so the time and cost of using robotic systems is reduced and finally the overall consultation time is reduced. One of the limitations of this approach is the high capacity need for storage of all images. Consequence of these hybrid systems was introducing a new field in pathology with terminology of virtual microscopy and virtual slides. In this technology a conventional prepared glass slide is placed on a motorized stage of a microscope with capacity of automatic focusing. The slide is scanned completely and consequently using all object lens and then these images are integrated to produce a single large image file [10-12]. This file can then be viewed in a computer in each location. In this method there is nor sampling error seen in static telepathology neither requirement to extensive equipment for distance control of microscope seen in dynamic telepathology. The only problem is the high capacity need for storing images (approximately 150 Mb) [13].

Despite the pervading use of telepathology in the world especially for first line diagnosis in area without on-site pathologist [14,15] it is not so popular in Iran and its use confined to a few centers, which used it only for consultation. There is no special network in Iran for telepathologic consultation and all of this centers use general web sites such as iPath. The aim of this study is to review the function of one of the most important of these centers in Iran and summarized the important problems which are limited the use of telepathology in Iran.

## Materials and methods

We analyzed our new experience in telepathologic consultation in cancer Institute of Tehran University of Medical Sciences using iPath telepathology server of Basel University. One hundred sixty one cases in a period of 32 month (March 2001 – December 2003) were evaluated. All of the cases in this study were referred for second evaluation to this centre but the definite diagnosis was not made here too. The representative microscopic H&E images along with other special technique images such as special staining, IHC, CT-Scan with history of the patients were uploaded to iPath server and after evaluation of comments a final diagnosis was made whenever possible. The microscope was used in this study was Carl Zeiss Axiolab and the digital camera was coolpix 2500 Nikon. All of the images had resolution of 1600 × 1200 pixels and the connection bandwidth was 100 Mbps. The diagnosis by clients and consultants are classified as benign, indeterminate, suspicious, and malignant. Demographic variables, number of comments, duration for final diagnosis, number of images and many other variables and correlation of specific final diagnosis with these variables were evaluated with SPSS.13 software.

## Results

**Figure 1:**
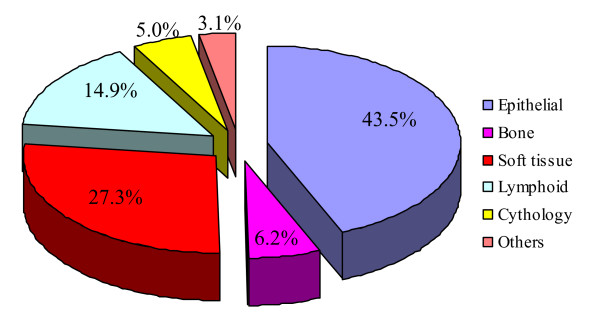
Percent of different neoplastic lesion, submitted for teleconsultation.

One hundred sixty one consultations (53% male, 47% female) were evaluated with average age of 39 years (range: 1–87). Eight of them were body fluids cytology and the remaining were neoplastic lesions of epithelial type, bone tissue, lymphoid tissue, soft tissue and other unknown lesions (figure 1).

**Figure 2:**
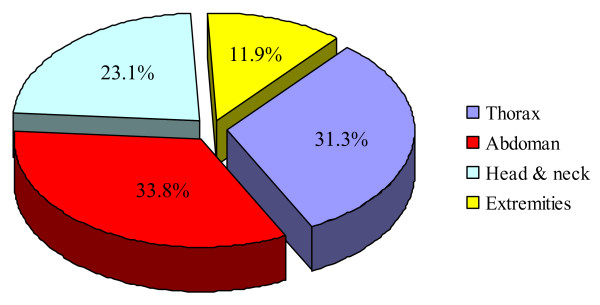
Location of teleconsulted neoplastic tissue.

The most location was abdominal cavity followed by thorax, head and neck and extremities (figure 2).

The average number of H&E images for each cases was 8 (range: 3–23, mode:5), 45 cases had immunohistochemical images and only 4 cases had other staining images, 4 cases also had accompanied radiologic images; 3 had clinical and 8 had gross pathologic images.

**Figure 3:**
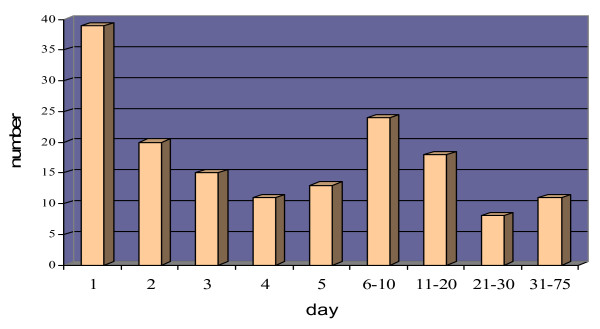
Number of the final diagnosis according to the days after submitting for consultation.

An average number of 2 (range: 1–7, mode: 2) consultants per each case presented their comments or diagnoses and the mean of comments for each case was 4 (rang: 1–14, mode: 3). In 51 cases the first comment was made in less than 8 hours (a single working day) and only in 9 cases (5.1%) the final diagnosis was reported in 8 hours (figure 3).

A diagnosis was differed in 1 case; among the others a definite final diagnosis with clinically unimportant discrepancies was achieved in 88 cases (55%). The comparison between the final diagnosis in these cases and the primary diagnosis of institute pathologists revealed 28% discrepancy between them which 8% were the cases with primary malignant diagnosis and final benign diagnosis. Seven benign and 2 malignant cases have been changed and the number of 16 cases with indeterminate and suspicious primary diagnosis was decreased to 5 after consultation (table 1). For 42 (26%) of the remaining 72 cases a recommendation to performing a specific procedure were propounded; and in 30 cases (19%) there were major discrepancies between consultant's opinion and so definite diagnosis was not made for them through teleconsultation. The primary diagnosis in these cases is shown in table 1.

**Table 1:** Comparison between primary diagnosis and final telepathologic result

	With final telepathologic diagnosis				Without final telepathologic diagnosis	
		
Primary diagnosis	Benign	Malignant	Suspicious	Indeterminate	No diagnosis	Further evaluation
Benign	14(16)*	2(2)	0	1(1)	4(6)*	4(6)
Malignant	7(8)	47(53)	0	1(1)	15(21)	10(14)
Suspicious	1(1)	3(3)	0	0	1(1)	1(1)
Indeterminate	3(3)	6(7)	1(1)	2(2)	10(14)	27(37)

*Numbers in parenthesis are in percent

There was neither relation between definite final diagnosis and anatomic location (*p *= 0.23) nor between definite final diagnosis and histology of the specimens (*p *= 0.42). The rate of achieving final diagnosis was not different between cases with IHC images and the others (*p *= 0.21). Increase in number of consultants has no effect on achieving a definite final diagnosis (*p *= 0.56) and there was no relation between number of H&E images and final diagnosis (*p *= 0.9). Categorizing the cases to cytological and histological groups revealed that the rate of achieving final diagnosis was higher in histological group (*p *= 0.01). The statement of inappropriate resolution and inappropriate selection of images were higher in cases without final definite diagnosis (*p *= 0.01), however more than 90% of consultants had not stated their opinions. Table 2 shows some details of findings of this study.

**Table 2:** Details of findings in different histological tumours, submitted for teleconsultation.

	**Soft tissue**	**Bone**	**Epithelial**	**Lymphoproliferative**	**Cytology**	**Other**
**IHC staining**	15	2	13	14	0	**1**
**Special staining**	1	1	1	1	1	**0**
**Comment for further evaluation**	14	13	10	10	5	**0**
**Definite telepathologic diagnosis**	25	5	42	10	1	**5**
**Without telepathologic diagnosis or comment**	5	2	17	4	2	**0**
**Final telepathologic diagnosis in less than 8 hours**	8	4	21	9	1	**1**

*Numbers in parenthesis are in percent

## Discussion

Use of telepathology for consultation is appearing to have many advantages over conventional light microscopy. The UICC has estimated that at least in 5–10% of cancer cases a pathologist need consultation during routine work because of uncertainty [16]. Sending glass slides or paraffin blocks by mail or courier for experts in the field, is a time consuming way especially in critical specimens for pathologists working alone in distant hospitals with no facilities for intradepartmental consultation. Besides, the probability of loss and damage are always present [17]. Today, telepathology in the forms of static and dynamic seems to be the basic solution for this major problem. However Mairinger et al report that only 15% to 44% of pathologists prefer to perform a consult through the images [18]. Along this viewpoint, other problems such as special equipments for taking digital images, a PC and a high bandwidth internet connection, which are not accessible in far areas, limit the use of telepathology despite the primary thriving view. The new method of virtual pathology shed line in the way of future telepathology. Conventional pathology with glass slide has many limitations. For example they may be easily broken, their stain is unstable and could fade with time, the tissue mount can bubble and dry out and finally certain procedure such as fluorescent stains are not stable more than few days. Dynamic and static telepathology also cannot eliminate all of these limitations because of their own limitations, which had been stated above. In this situation it seems that the best replacement for conventional slide pathology is virtual slides, which never change in appearance as long as the data integrity is maintained. Its coast is much less than dynamic telepathology, and the need for broadband connections for transmission could be solved by batched overnight transmission since simultaneous link is not required for initial examination [13]. It is also a good approach for tele-education and already is used widely for this purpose. However in the spite of mentioned points, telepathology in Iran is not popular and Except Mirskandari's study [19] there is no documented study in Iran about telepathology. In this study we wanted to evaluate the position of telepathology in Iran. This is used only for consultation in limited centers and only in the form of static telepathology because of the limitations in equipments and in bandwidth of internet connections. Three is also no special network in Iran for this purpose. Cancer institute is the first centre that began telepathologic consultation through iPath server. The findings of our study are so far from other similar studies. Brauchli et al reported 94% definite final diagnosis [20] and Desai et al in two separate studies reported 93% and 90% achieving final diagnosis respectively [21,22] in contrast with 55% in our study. In other controlled trials the accuracy of static telepathology was more than 85% [23-25]. However in cases which final diagnosis was made for them by teleconsultation (88 cases) grate improvement was seen such as decrease in the number of suspicious and indeterminate cases from 16 to 5 which is very important in clinical approach. Table 1 also shows that 8 benign and 3 malignant cases are changed after consultation. These changes are very critical because could completely change the approach to the patient, but unfortunately we have no follow up to evaluate these findings. It shows that in this group teleconsultation has grate advantage. Table 1 shows that 53% of the cases in which final diagnosis was not made after consultations are ones that had indeterminate or suspicious primary diagnosis.

In our study only in 5% the final diagnosis made in a single working day in comparison with 32% in Desai's studies [21,22]. Major discrepancies were seen in 6% and 9% of the cases in Desai's studies, but it is 19% in our study. In contrast to our expectation there was no association between numbers of H&E images and number of consultants with definite final diagnosis. More than 90% of consultants had no idea about the quality of the images and make their opinions, so we regarded that our images quality was good. Besides in other studies it is stated that minimum resolution is required for teleconsultation is 1024*768 pixels and it was 1600*1200 pixels in our study [23].

The explanation for these significant differences with other studies is rather simple. As formerly pointed, cancer institute is a referral centre with expert pathologists, and the cases which selected for telepathologic consultation were cases that institute's pathologist had some problem to making definite diagnosis for them in primary consultation. In the other word these cases are problematic ones which many studies stated that are not suitable for telepathologic consultation, because in many of them paraffin blocks are needed for more specific evaluation [26,13] as it occur in 26% of our cases. The other possible reason which is stated in other studies as the main reason of low level of accuracy in static telepathology may be image sampling error. It could be very significant in this study because of little experience in telepathology in Iran. In our study the rate of achieving final diagnosis in cytological specimens is lower than histological ones like other studies [27-29]. This is because of the essence of cytology which requires a vigilant search of the whole slide and so it is very sensitive to undersampling by static telepathology. The other probable reason which should be regarded not also in this study but in other similar studies for discrepancies in final diagnosis is the different use of medical terms in one situation, we could not evaluate it in this study but we offer to use numeric based classification system such as ICD-O code in telepathologic software beside other parameter in their unit platform to get more comparable results.

A brief attention to the above mentioned points reveals the first steps toward the use of telepathology in Iran. Because of the large distance between some rural and referral centres in Iran, consulting with an expert pathologist is one of the difficult, time consuming and expensive affairs. Regarding the limitation of internet bandwidth connection in Iran and no access to special equipment in many remote areas it seems that static telepathology is the only form of telepathology which now could be used. The relatively low rate of accuracy of static telepathology is the most important reason for not accepting of this method in routine works. The accuracy of static telepathology has been reported to be range from 68% to 95% [30] in comparison with 95% to 100% in dynamic telepathology [31,32]. But there are isolated reports of 95% to 100% accuracy [33]. This wide rang of accuracy results from the different interpretation, video image quality, video monitor experience, and most importantly from field selection [30,34-36]. Field selection is the most important reason that account for low accuracy and is the main reason which was stated with pathologist for low rate of telepathology acceptance in Mairinger's study [18]. However it is proved in many studies that this problem could be significantly improved with training, description of well-defined protocols for sampling of each specific specimen [13,37]. There is also evidence that over time this problem decreased with increasing experience [38]. Thus it seems that education and clear guidelines for pathologist is essential before starting static telepathologic network. Virtual pathology is the most important thing should be taking into account in the feature planning. Because beside the above mentioned benefits it has no sampling error and all the things it needs are a microscope with automatic motoralized stage and a digital camera [11] which are not so expensive if we regard the cost of conventional consultation used in Iran. The problem of limited bandwidth of connections in Iran is also could be overcome with overnight transmission since dynamic links are not required [13]. It is also could be used in complex centers like cancer institutes with multiple pathologic wards, eliminating the need for time consuming search of staff and sorting of glass slides. The problem of connection bandwidth is also solved by designing an internal network.

Finally we should say that this form of telepathology that experienced in Iran is not accurate. The best way seems to be designing a software compatible with Iran network characteristics to connect small rural centres to referral ones to performing the present numerous requests for consultation and subsequently saving time and money. It needs preparing specific equipments for rural centres, designing special software, efficient network connection and finally continuous education of pathologist to change their opinion about use of static telepathology in their routine works and taking to account the benefits of replacing conventional pathology by virtual pathology.
